# Rally to the Standard

**Published:** 1894-11

**Authors:** 


### EDITORIAL DEPARTMENT.

F. E. DANIEL, M. D., Editor.
S. E. HUDSON, M. D., Managing Editor.
A. J. SMITH. M. D., Galveston, Associate Editor.

EDITORIAL STAFF:

PROF. J. E. THOMPSON, M. D., Texas Medical College, Galveston; Surgery.
PROF. WM. KEILLER, M. D., Texas Medical College, Galveston; Obstetrics and Gynecology.
PROF. DAVID CERNA, M. D., Texas Medical College, Galveston; Therapeutics.
PROF. A. J. SMITH, M. D., Texas Medical College, Galveston; Medicine
DR. ISADORE DYER, Tulane University; Dermatology.
DR. R. H. L. BIBB, Saltillo, Mexico; Foreign Correspondent.

Official organ of the West Texas Medical Association, the Houston District Medical Association, the Austin District Medical Society, the Galveston County Medical Society, and several others.

### RALLY TO THE STANDARD.

Attention is asked to the letter from Dr. Wooten, published elsewhere in this issue.

Dr. Wooten, in one sense, may be said to be the father of the Texas University,—certainly he has been the prime mover in securing the establishment of a medical branch, and has been indefatigable in its interest. His devotion to the cause has endeared him very much to the medical profession, and his opinion on any subject connected with it, or upon anything that concerns its welfare, should and does receive respectful consideration.

The doctor points out the absurdity of the State permitting, by its lax laws, half educated or not at all educated persons to come into the State to practice, when she has founded a home school and requires a high order of ability on the part of her sons to entitle them to practice; to allow the refuse of other States to come in competition with her own graduates. It is unreasonable and unjust, and although, so far, every effort at a change in our medical act has been a failure, Dr. Wooten has not lost hope, but calls again upon the influential members of the profession throughout the State to aid him in securing the passage of a bill that will remedy the evil. If every physician in Texas who has a proper pride in his profession would personally appeal to the immediate representative from his county, and present the matter in its proper light, there is reason to hope that, notwithstanding our past failures, we may be able to get a good law; such a law as is in operation in other States. It is a reproach to Texas that she has no medical law. There is practically no restrictions placed upon the privilege; anybody, from anywhere, who can show a diploma of any kind, can, by registering that diploma with the clerk of the county court, practice medicine.

The JOURNAL has had so much to say upon this subject for the past ten years, that it seems almost threadbare; but it has lost none of its interest, however well nigh despairing; and a renewed effort, inaugurated by the venerable President of the University Regents, kindles afresh our zeal, and we, for our part, will lend him all the aid in our power.

The opposition to medical reform has heretofore come from the homeopaths; but the plan suggested by Dr. Wooten surely can awaken no opposition, either from them or from the eclectics.

Dr. Wooten proposes to have printed a list of all the medical colleges which require three or more years graded course, and certain other requirements, as condition precedent to granting a diploma, and to furnish that list to every county court in Texas. He will ask for a bill which stipulates that the privilege of practicing medicine in Texas shall be granted to only those who hold such diploma, and as this list will embrace homeopathic and eclectic colleges as well as the regular, it would seem that all opposition would be disarmed, and there could be no objection to its passage. This is especially true because the status of the long-time practitioner, whether a graduate or not, and of all who have become, under prior or existing laws, legally entitled to practice, will not be disturbed.

In other words, we must accept the evil as it is; let alone all who are now practicing, if legally, but put up the bars against all comers in future who are turned loose half qualified.

The only difference will be, should Dr. Wooten's idea materialize, that there will be discrimination in registering diplomas; now there is none; and the absurdity of licensing a first course student will be done away with. The desired object can be accomplished by a very short amendment to our present law; it will not be necessary to get up a lengthy bill, like those we have heretofore presented.

The JOURNAL hopes all live medical men in the State will canvass the subject with their representatives and senators, and secure the promise of their support for the measure. If necessary, make such promise a condition to support of the candidate. Our next legislature must give us a better law; the interest of the public, justice, everything demands it.

